# The impact of human hyperekplexia mutations on glycine receptor structure and function

**DOI:** 10.1186/1756-6606-7-2

**Published:** 2014-01-09

**Authors:** Anna Bode, Joseph W Lynch

**Affiliations:** 1 Queensland Brain Institute, The University of Queensland, Queensland 4072, Australia; 2 Queensland Brain Institute and School of Biomedical Sciences, The University of Queensland, Queensland 4072, Australia

**Keywords:** Cys-loop receptor, Ligand-gated ion channel, Chloride channel, Startle disease, Glycinergic neurotransmission

## Abstract

Hyperekplexia is a rare neurological disorder characterized by neonatal hypertonia, exaggerated startle responses to unexpected stimuli and a variable incidence of apnoea, intellectual disability and delays in speech acquisition. The majority of motor defects are successfully treated by clonazepam. Hyperekplexia is caused by hereditary mutations that disrupt the functioning of inhibitory glycinergic synapses in neuromotor pathways of the spinal cord and brainstem. The human glycine receptor α1 and β subunits, which predominate at these synapses, are the major targets of mutations. International genetic screening programs, that together have analysed several hundred probands, have recently generated a clear picture of genotype-phenotype correlations and the prevalence of different categories of hyperekplexia mutations. Focusing largely on this new information, this review seeks to summarise the effects of mutations on glycine receptor structure and function and how these functional alterations lead to hyperekplexia.

## Hyperekplexia and glycine receptors

**Figure 1:**
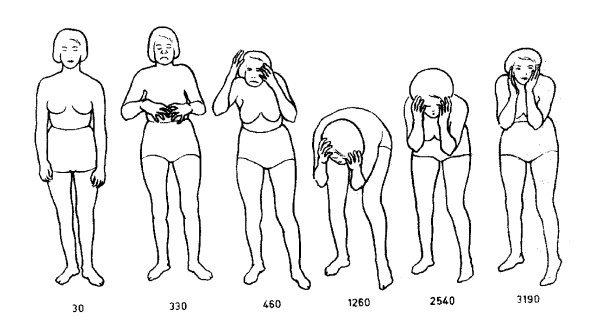
**Schematic of a hyperekplexia patient illustrating the sequence of movements during a startle reflex.** Numbers represent elapsed time in ms. Reproduced with permission from Elsevier [2].

Hyperekplexia (OMIM #149400), or human startle disease, was first reported in 1958 by Kirstein and Silfverskiold [1]. They described a family in which four members suffered sudden falls precipitated by ‘emotional’ stimuli including surprise, fear or stress. In 1966, Suhren and colleagues reported similar symptoms in a larger pedigree that were shown to be inherited in an autosomal dominant manner and were treatable by barbiturates [2]. We now know that hyperekplexia is a rare neurological disorder characterized by 1) episodic and generalized stiffness after birth which gradually subsides during the first years of life, 2) an increased likelihood of apnoea attacks, delayed speech acquisition and/or intellectual disability, 3) excessive startle reflexes to unexpected stimuli, particularly auditory or tactile, that persist throughout life, and 4) a transient generalized stiffness after startle reflexes that can result in injurious falls [3-6]. The classic startle response is characterized by forceful closure of eyes, rising of bent arms over the head and flexion of the neck, trunk, elbows, hips and knees (Figure 1). Consciousness is fully retained during these episodes, thus distinguishing hyperekplexia from epileptic seizures. Clonazepam, a benzodiazepine that positively modulates inhibitory synaptic gamma aminobutyric acid (GABA) type-A receptor chloride channels, is a highly effective treatment [3].

Shiang and colleagues were first to show that hyperekplexia is caused by hereditary mutations in the *GLRA1* gene that encodes the α1 subunit of the inhibitory human glycine receptor (hGlyR) chloride channel [7]. Although the *GLRA1* gene represents the major gene of effect [8,9], hyperekplexia can also be caused by mutations in the *GLRB* gene which encodes the hGlyR β subunit [10-15] or in the *SLC6A5* gene which encodes the presynaptic glycine transporter type-2 [16-19]. Mutations have also been identified in the genes encoding the hGlyR synaptic clustering proteins, gephyrin [20] and collybistin [21]. However, the phenotypes resulting from the later mutations are more complex than the classical phenotype described above. The common feature of all these proteins is that they are required for the normal functioning of inhibitory glycinergic synapses. As a large proportion of hyperekplexia cases have no genetic explanation [4], the analysis of other proteins involved in the development or function of glycinergic synapses could reveal further susceptibility genes. At this stage, however, the proteomics of glycinergic synapses is not well understood.

Inhibitory glycinergic synapses are located predominantly in the spinal cord and brainstem [22-24] and disruptions to their function increase the general level of excitability of motor neurons, thus accounting for neonatal hypertonia. Although patients seem to develop compensatory mechanisms to cope with this chronically enhanced excitability, they are not able to deal with the increased inhibitory demand required to dampen strong, unexpected excitatory commands [3].

Recently, thanks to large-scale systematic genetic screening programs, several hundred hyperekplexia probands have been examined and the results have generated a clear picture of the type and prevalence of mutations, their inheritance modes and the mechanisms by which they affect hGlyR structure and function [8,9,11,12,25]. The field may thus be considered to have reached a state of maturity. The clinical presentation and genotype-phenotype correlations in hyperekplexia have recently been published [4,5,26]. The aim of this review is to summarise the effects of mutations on hGlyR structure and function and how these functional alterations lead to hyperekplexia.

## Molecular structure, stoichiometry and expression of GlyRs

**Figure 2:**
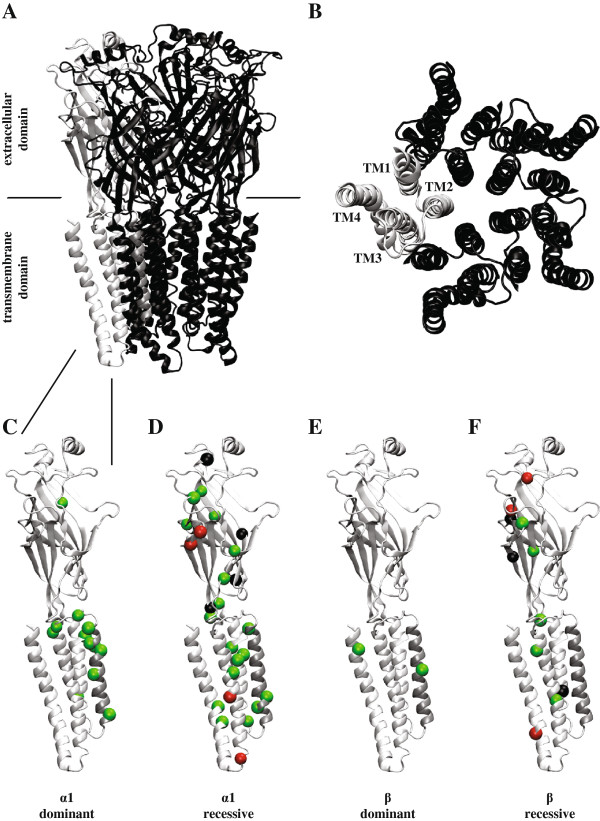
**pLGIC structure and the locations of hGlyR hyperekplexia mutations.** The top panel shows the pentameric structure of the *C. elegans* α glutamate-gated chloride channel receptor (PDB 3RIF [31]) viewed from within the membrane **(A)** and from the presynaptic terminal **(B)**. One subunit is coloured light grey. Panels **C-F** show a single pLGIC subunit with the locations of dominant and recessive mutations in the α1 and β hGlyR subunits coloured in green (missense), red (nonsense) or black (deletions). TM2 is coloured dark grey.

GlyRs belong to the Cys-loop family of pentameric ligand-gated ion channel (pLGIC) receptors. The X-ray molecular structures of several pLGIC receptors have been solved, including two bacterial homologues crystallized in the closed and open states, respectively [27-29], a nicotinic acetylcholine receptor (nAChR) from *T. marmorata*[30] and a glutamate-gated chloride channel receptor from *C. elegans*[31]. As this later receptor exhibits ~34% sequence homology with the α1 hGlyR subunit, it provides an excellent molecular structural template. As shown in Figure 2A, B GlyRs consist of five subunits arranged symmetrically in a ring around a central ion-conducting pore. Each subunit contains an extracellular domain (ECD), comprised mainly of a twisted β-sheet sandwich, harbouring the ligand binding site, and a transmembrane domain (TMD) comprising four α-helices, termed TM1 – TM4, with the five TM2 domains lining the axial channel pore. Both the extracellular β-sheets and transmembrane α-helices are connected by flexible loops. Glycine binds at the extracellular subunit interface and maximum gating efficacy is reached when three of the five binding sites are occupied [32]. Upon glycine binding, each ECD rotates relative to its TMD, ultimately inducing an outward tilt of the top of the TM2 domains which then opens the pore [33-35].

In humans, four α subunits (α1 – α4) and a single β subunit have been described. The human α4 subunit is considered a pseudogene on the grounds that it incorporates a premature stop codon upstream of the final TM4 domain [36]. hGlyRs exist either as α homomers or as αβ heteromers in a stoichiometry of 2α:3β [37,38] or 3α:2β [39]. As the β subunit mediates hGlyR attachment to the sub-synaptic clustering protein, gephyrin [40], it is presumed that only heteromeric αβ hGlyRs are present in synapses. Embryonic rats express predominantly α2 subunits, with the onset of β subunit expression coinciding with the first appearance of glycinergic synapses around the time of birth [41]. By three weeks postnatal in the rat spinal cord, most α2 subunits have been replaced by α1 subunits. In the adult spinal cord, α1 and β subunits are expressed in glycinergic synapses in motor reflex arcs, whereas α1, α3 and β subunits are all expressed in inhibitory synapses in pain sensory neurons in the superficial laminae of the dorsal horn [42]. In the cerebral cortex and hippocampus, GlyRs are extra-synaptic only and comprise predominantly α2 homomers or α2β heteromers [43-46]. This expression pattern implies that α1 and β subunits should be targets of hyperekplexia mutations whereas α2 and α3 subunits should not.

## Hyperekplexia mutations in the hGlyR α1 subunit

### Relationship between mutation type and inheritance mode

Most α1 hGlyR hyperekplexia mutations are either missense mutations whereby a single nucleotide change results in a codon change for a different amino acid, or nonsense mutations whereby a single nucleotide change leads to a premature stop codon (Table 1). Large deletions are also found, especially deletions of exons 1 – 7 in families of Turkish origin, suggesting that this is a population-specific risk-allele [9]. Hyperekplexia mutations can be inherited in both autosomal dominant or recessive modes with the majority of mutations being recessive (Table 1). Recessive mutations can be homozygous recessive, as first reported in 1994 [47] or compound heterozygous, as first described in 1999 [48]. Mutations can also be *de novo* meaning that neither parent possesses the mutation [49]. To date, there is no evidence for a correlation between clinical traits and inheritance mode of *GLRA1* mutations [4].

**Table 1:** **Hyperekplexia mutations in** ***GLRA1***

**Mutation**	**Mutation type**	**Inheritance**	**hGlyR position**	**Notes**	**Reference**
ΔEx1-7	deletion	recessive	n.a.		[50]
ΔEx4-7	deletion	recessive	n.a.	compound heterozygous with R65L	[9]
R65L	missense	recessive	ECD	compound heterozygous with ΔEx4-7	[9]
R65W	missense	recessive	ECD	compound heterozygous with P230S	[9]
W68C	missense	recessive	ECD	compound heterozygous with R316X	[51]
R72fsX47	deletion	recessive	ECD		[52]
R72H	missense	recessive	ECD		[53]
R72C	missense	recessive	ECD		[8]
E103K	missense	recessive	ECD	compound heterozygous with L184fsX21	[9]
Y128C	missense	dominant	ECD		[9]
K132fsX15	deletion	recessive	ECD		[54]
C138S	missense	recessive	ECD	compound heterozygous with D148fsX16	[55]
M147V	missense	recessive	ECD		[52]
D148fsX16	deletion	recessive	ECD	compound heterozygous with C138S	[55]
D165G	missense	recessive	ECD		[9]
W170S	missense	recessive	ECD		[56]
L184fsX21	deletion	recessive	ECD	compound heterozygous with E103K	[9]
Y197X	nonsense	recessive	ECD	compound heterozygous with Y202X	[9]
Y202X	nonsense	recessive	ECD	compound heterozygous with Y197X	[52]
R218Q	missense	*de novo*	ECD	compound heterozygous with S296X	[49]
R218W	missense	recessive	ECD		[8]
Q226E	missense	dominant	TM1		[8]
Y228C	missense	recessive	TM1		[57]
P230S	missense	recessive	TM1	compound heterozygous with R65W	[8]
S231R	missense	recessive	TM1		[58]
S231N	missense	recessive	TM1	compound heterozygous with S296X	[9]
W239C	missense	dominant	TM1		[59]
I244N	missense	recessive	TM1		[47]
P250T	missense	dominant	TM1-TM2 loop		[60]
R252H	missense	recessive	TM2	compound heterozygous with R392H	[48]
R252C	missense	recessive	TM2		[9]
G254D	missense	recessive	TM2		[9]
V260M	missense	dominant	TM2		[49]
T265I	missense	dominant	TM2		[9]
Q266H	missense	dominant	TM2		[61]
S267N	missense	dominant	TM2		[62]
S270T	missense	recessive	TM2		[63]
R271L	missense	dominant	TM2		[7]
R271Q	missense	dominant	TM2		[7]
R271P	missense	dominant	TM2		[64]
R271X	nonsense	dominant	TM2		[13]
K276E	missense	dominant	TM2-TM3 loop		[65]
K276Q	missense	*de novo*	TM2-TM3 loop		[66]
Y279C	missense	dominant	TM2-TM3 loop		[67]
Y279X	nonsense	recessive	TM2-TM3 loop		[59]
Y279S	missense	dominant	TM2-TM3 loop		[68]
V280M	missense	dominant	TM2-TM3 loop		[8]
L291P	missense	recessive	TM3	compound heterozygous with D388A	[8]
S296X	nonsense	recessive	TM3	compound heterozygous with S231N and R218Q	[69]
R316X	nonsense	recessive	TM3-TM4 loop	compound heterozygous with W68C	[51]
G342S	missense	recessive	TM3-TM4 loop		[70]
E375X	nonsense	recessive	TM3-TM4 loop		[8]
D388A	missense	recessive	TM3-TM4 loop	compound heterozygous with L291P	[8]
R392H	missense	recessive	TM4	compound heterozygous with R252H	[48]
R414H	missense	dominant	TM4		[8]

ECD extracellular binding domain, TM transmembrane.

*GLRA1* nonsense and deletion/frameshift mutations, which lead to a loss of protein expression at the cell surface, are invariably autosomal recessive (Table 1). The reason for this is that the unaffected allele can generate sufficient quantities of protein to support normal glycinergic neurotransmission. In contrast, autosomal dominant mutations are missense mutations and invariably express strongly in cell surface-expressed hGlyRs, but diminish GlyR current-carrying capacity via spontaneous channel activity or via reductions in glycine sensitivity, zinc sensitivity, open probability and/or single channel conductance. Due to the efficient expression of these mutated subunits, their deleterious effects cannot be rescued by the unaffected allele. Recessive mutations are scattered throughout the α1 hGlyR subunit while dominant mutations are clustered in and around the pore-lining TM2 domain (Figure 2C, D).

Here, we describe the effects of those mutations (mainly autosomal dominant) that provide useful insights into the structure and function of hGlyRs and/or the pathophysiological mechanisms of hyperekplexia.

### Spontaneous activation

So far, four *GLRA1* mutations resulting in spontaneous channel activity have been identified: Y128C [9], Q226E, V280M and R414H [8]. All four mutations are autosomal dominant and the mutated subunits express strongly. Some possible mechanisms by which spontaneous hGlyR activation may give rise to hyperekplexia are considered below.

**Figure 3:**
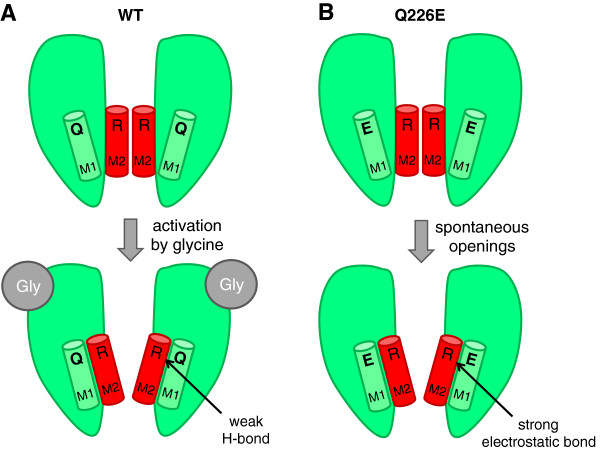
**Proposed mechanism by which Q226E induces spontaneous activation.** The TM1 and TM2 helices are coloured green and red, respectively, and are located in adjacent subunits. **A**. In the wild type (WT) α1 hGlyR, glycine induces activation by tilting the top of TM2 away from the pore axis towards TM1, where the open state is weakly stabilized by an H-bond between Q226 and R271. Hyperekplexia mutations at R271 are likely to disrupt this bond, thus destabilising the open state. **B**. In the Q226E mutant α1 hGlyR, a stable open state in the absence of glycine is induced via the formation of a strong electrostatic bond between Q226E and R271.

Q226E, located at the top of the TM1 domain (Figure 3A, B), also produces modest reductions in single channel conductance and cell surface expression efficiency that may contribute to the hyperekplexia phenotype [8]. Recent functional evidence suggests that Q226E induces receptor activation via an enhanced electrostatic attraction to R271 located at the top of the TM2 domain in the neighbouring subunit [25]. This attraction would tilt the top of the TM2 domain away from the pore axis, towards the TM1 domain, to constitutively open the channel. As detailed below, R271 is also an important hyperekplexia locus.

V280M, in the TM2-TM3 loop, exhibits a dramatically enhanced glycine sensitivity and spontaneous channel activity suggesting a drastic destabilization of the closed channel state [8]. We propose that the increased side chain volume of V280M exerts a steric repulsion against I225 at the top of the TM1 domain in the neighbouring subunit [25]. This would tilt the top of the TM3 domain radially outwards against the stationary TM1 domain and thus provide space for the TM2 domain to relax away from the pore axis to create an open channel.

Y128C is located in the inner β-sheet of the ECD. The mechanism by which it induces spontaneous activity is not yet resolved, but given its distance from the TMD, it seems likely that it causes non-specific structural alterations [9].

R414H, in the TM4 domain, results in a very low rate of spontaneous activity and has weak, if any, effects on glycine sensitivity, single channel conductance and expression efficiency [8]. It is thus unclear how this mutation causes hyperekplexia. As R414H has recently been identified as a rare single nucleotide polymorphism, it may not actually be responsible for hyperekplexia in the affected individual.

The high level of spontaneous activity in the Y128C, Q226E and V280M mutant hGlyRs directly contributes to the observed reduction in the glycine-induced current amplitude [8,9]. The tonic chloride influx may also shift the chloride equilibrium potential to more positive values leading to a further reduction in the inhibitory efficacy of glycinergic neurotransmission or even a chronic depolarization that could lead to an increased action potential firing rate. In addition to directly activating neurons, tonic hGlyR activation could result in enhanced sodium and calcium influx rates. The effects of the mutations could thus be similar to those of nAChR slow-channel myasthenia mutations that result in ‘cationic overload’ of the postsynaptic region which destroys synaptic specializations and intracellular organelles [71]. As discussed below, a hyperekplexia mutation (L285R) in the hGlyR β subunit also causes spontaneous channel activity.

### Impaired channel gating

R271Q and R271L, at the extracellular end of the TM2 domain, are the most frequently occurring and the most studied hyperekplexia mutations. They are both inherited in an autosomal dominant manner [7,47,65,67,72]. A rare autosomal dominant mutation at this site, R271P, is yet to be functionally characterized [64]. R271Q and R271L do not impair cell surface expression but dramatically reduce both the glycine sensitivity and the single channel conductance [73-77]. The decrease in single channel conductance most likely results from the elimination of the positive charge on R271. This would diminish the ability of the pore to concentrate chloride ions in its external vestibule, which would in turn reduce the chloride influx rate [25,78]. Unfortunately, however, the effects of R271 mutations on glycine sensitivity do not have such a simple molecular explanation.

The TM2-TM3 loop located adjacent to R271 is an important structural element involved in transmitting glycine binding signals from the binding site to the activation gate [33-35]. Given that this is achieved via a highly organized network of energetic interactions between residues in the TM2-TM3 loop and the ECD, any alteration to loop structure would be expected to impair the efficient gating of the receptor, leading to reductions in glycine sensitivity and maximum open probability [79]. Evidence to date suggests that all hyperekplexia missense mutations in this loop, including R271Q/L/P, K276E/Q and Y279C/S, impair hGlyR function via a similar mechanism [74,75,79-81]. As illustrated in Figure 3, one specific effect of the R271 mutations is to disrupt a hydrogen bond with Q226 that is required to stabilise the open state [25]. Without this hydrogen bond, the open state would be destabilized, thereby reducing glycine sensitivity.

These and other mutations that impair channel gating cause hyperekplexia by reducing the rate of chloride influx through synaptic hGlyRs. Depending on the mutation, this may be achieved via a combination of a reduced maximum channel open probability, a reduced single channel conductance and/or a reduced the glycine sensitivity which would diminish the likelihood of the channels being effectively activated by synaptic glycine concentrations. The consequent reduction in glycinergic current magnitudes would disinhibit motor neurons thereby leading to enhanced firing activity and more potent muscle contractions. As noted above, hyperekplexia patients develop compensatory mechanisms to cope with the level of excitatory activity required for normal motor control [3]. One compensatory mechanism, identified in mouse models of hyperekplexia, is an enhancement of inhibitory GABAergic neutrotransmission [82], which may explain why clonazepam is an effective treatment. However, during startle episodes, the weakened inhibitory system is unable to dampen the excessive level of excitatory activity in motor neurons and the classic startle response results.

Although hyperekplexia patients with R271Q/L mutations are effectively treated with clonazepam [3,4,6], novel alternate therapeutic strategies are emerging. In one recent study, hGlyR function was restored by shifting the R271Q/L residue out of the allosteric signalling pathway via the mutation of surrounding residues [83]. This result raises the possibility of either designing or re-purposing drugs that bind in the alcohol/anaesthetic site near to R271 to achieve the same outcome [84]. In addition, the anaesthetic and GlyR positive allosteric modulator, propofol, preferentially enhances the potency of glycine in R271Q/L relative to α1 wild type hGlyRs [85,86], and indeed, propofol successfully normalized hyperekplexia symptoms in a mouse carrying the R271Q mutation [86]. As propofol binds in the deep cleft near R271 [87], it offers a starting point for identifying novel, more specific hyperekplexia treatments. Although the development of new drugs to treat specific hyperekplexia genotypes is unlikely to be economically viable, the re-purposing of existing clinically-approved drugs may be a realistic option.

The autosomal dominant mutation Q266H [61] in the TM2 domain reduces glycine sensitivity and single channel open times indicating that it also disrupts receptor gating efficacy [88]. Autosomal dominant hyperekplexia mutations to other pore-lining residues, V260M, T265I and S267N, similarly disrupt glycine efficacy [9,62,89]. Interestingly, the S267N mutation also abolishes hGlyR ethanol sensitivity [62], although the sensitivity of the patient to alcohol was not reported.

The autosomal dominant mutation R218Q in the ECD produces a dramatically reduced sensitivity to glycine, which is probably the primary reason for its hyperekplexia phenotype [49,89]. Low concentrations of the competitive antagonist strychnine were similarly antagonized in wild type and R218Q-containing receptors suggesting that residue R218 plays an important role in channel gating, with only minor effects on glycine binding [89]. Recently, it was shown that the R218Q mutation disrupts a salt bridge between R218 and N148 that is crucial for efficient gating [90].

### Increased desensitization rate

The autosomal dominant P250T mutation in the TM1-TM2 loop reduces glycine-activated current amplitudes and induces fast desensitization with a time constant near 120 ms [60,91]. The reduced whole-cell current amplitude can be explained by a dramatic reduction in single channel conductance from around 80 to 1.3 pS, which no doubt accounts for much of the hyperekplexia phenotype [60]. In contrast, glycine sensitivity, affinity for strychnine and cell surface expression were similar to wild type receptors. Mutagenesis screening of neighbouring residues in the TM1-TM2 loop demonstrated that P250 is by far the most critical residue with respect to desensitization and glycine sensitivity [91]. Molecular dynamics simulations revealed an increased flexibility in P250T mutant hGlyRs which would destabilize the open state and explain the observed rapid desensitization [92]. The same publication also suggested that receptor activation and desensitization are structurally distinct processes as recently supported by a voltage-clamp fluorometry study [93]. As glycinergic synaptic currents exhibit decay time constants of 5 – 10 ms, it is unclear whether the enhanced desensitization rate induced by P250T is sufficient to limit chloride flux through the mutant channels and thereby contribute to the hyperekplexia phenotype.

The P230S hyperekplexia mutation in the TM1 domain also induces fast desensitization with a time constant near 1 s [8]. Additionally, this mutation reduces glycine sensitivity and maximal glycine-induced current amplitudes independent of β subunit co-expression. Genetic analysis of the patient with the P230S mutation suggested possible heterozygosity with R65W, although parental DNA was not available to confirm this. However, given the severity of the functional deficits resulting from the P230S mutation, we speculate it was most likely autosomal dominant. The R65W mutation eliminates cell surface expression and results in a recessive form of hyperekplexia [9].

### Reduced cell surface expression

Many hyperekplexia mutations reduce cell surface expression, thereby reducing the maximal glycine-induced current amplitude. For the autosomal recessive mutations, S231R and I244N, both located in the TM1 domain, R252H in the TM2 domain and R392H in the TM4 domain, it was shown that treatment with the proteasome blocker lactacystin significantly increased the accumulation of mutated α1 subunits in intracellular membranes suggesting that the mutated subunits were recognized by the endoplasmatic reticulum control system and then degraded via the proteasome pathway [94]. Thus, the loss of glycinergic inhibition associated with many recessive hyperekplexia phenotypes may be due to the sequestration of mutated subunits within the endoplasmatic reticulum quality control system.

hGlyRs incorporating premature stop codons usually do not form functional receptors at the cell surface [9,69,95]. However, it has recently been shown that the autosomal recessive truncation mutation, E375X, which truncates the α1 hGlyR upstream of the TM4 domain, can be incorporated into functional receptors together with α1 wild type subunits [8]. hGlyRs containing the truncated subunit exhibited low cell surface expression and reduced glycine sensitivity. As this truncation occurs upstream of the naturally occurring premature stop codon in the human *GLRA4* gene, it suggests that a review of the presumed pseudogene status of *GLRA4*[24,36] and of other similarly classified pLGIC genes may be warranted.

### Loss of zinc potentiation

Low concentrations of zinc (0.01 - 10 μM) have long been known to potentiate hGlyRs [96]. As zinc is concentrated in presynaptic terminals in the spinal cord and is released upon neuronal stimulation, its potentiating effect on glycinergic currents is likely to be physiologically relevant. Indeed, hyperekplexia symptoms were present in a genetically-modified mouse harbouring a mutant (D80A) α1 subunit that abolished zinc potentiation [97]. Recently, the *GLRA1* V170S mutation was shown to produce an autosomal dominant form of hyperekplexia [56]. When these mutant receptors were recombinantly expressed in a mammalian cell line, V170S was found to have no effect on glycine sensitivity although it completely eliminated zinc potentiation [98]. This result implies that hyperekplexia can result from a reduction in glycinergic current magnitude due to the elimination of zinc potentiation.

## Hyperekplexia mutations in the hGlyR β subunit

The *GLRB* gene has only recently been identified as a major gene of effect in hyperekplexia [10-12] although the first *GLRB* hyperekplexia mutation was identified in 2002 [15]. To date, one autosomal dominant mutation, Y470C, is known [11], although the *de novo* L285R substitution is also likely autosomal dominant given the nature of its effect on receptor function (see below) and the fact that it was identified in a heterozygous proband [12]. The remaining mutations are autosomal recessive as either homozygous recessive or as compound heterozygous (Table 2). Around half of these mutations result in the excision of large amounts of β subunit protein which would most certainly eliminate functional expression. Most of the remaining mutations (P169L, M177R, G229D, △S262, W310C, R450X, Y470C) either reduce cell surface expression of functional heteromeric hGlyRs and/or cause modest reductions in glycine sensitivity [11,12,15], both of which are typical effects of recessive mutations. Although molecular modelling has provided insight into possible structural defects caused by these mutations [11,12], experimental support for most of the model predictions is lacking to date.

**Table 2:** **Hyperekplexia mutations in** ***GLRB***

**Mutation**	**Mutation type**	**Inheritance**	**GlyR position**	**Notes**	**Reference**
ΔEx1-8	deletion	recessive	n.a.		[11]
Splice site mutation In4 (c.298-1G > A)	missense	recessive	n.a.	compound heterozygous with S321F	[13]
ΔEx5	deletion	recessive	n.a.	compound heterozygous with G229D	[15]
ΔEx5 and S176RfsX6	deletion	recessive	n.a.		[11]
E24X	nonsense	recessive	ECD		[11]
R50X	nonsense	recessive	ECD	compound heterozygous with Q216fsX222	[14]
P169L	missense	recessive	ECD		[11]
M177R	missense	recessive	ECD		[10]
R190X	nonsense	recessive	ECD	compound heterozygous with △S262	[11]
F19IfsX3	deletion	recessive	ECD		[11]
Q216fsX222	deletion	recessive	ECD	compound heterozygous with E24X	[14]
G229D	missense	recessive	ECD	compound heterozygous with ΔEx5	[15]
△S262	deletion	recessive	TM1	compound heterozygous with R190X	[11]
L285R	missense	*de novo*	TM2		[12]
W310C	missense	recessive	TM2-TM3 loop		[12]
S321F	missense	recessive	TM3	compound heterozygous with In4 (c.298-1G > A)	[13]
R450X	nonsense	recessive	TM3-TM4 loop		[11]
Y470C	missense	dominant	TM4		[11]

ECD extracellular binding domain, TM transmembrane.

The *de novo* mutation, L285R, provides an exception to the above pattern of effect on the grounds that it produces spontaneous channel activity when co-expressed with α1 wild type hGlyR subunits [11,12], and because structural basis of its defect can be inferred with confidence. L285 is located at the 9′ position in the middle of the pore-lining TM2 domain [30]. It has long been recognized that 9′ leucines are very highly conserved among pLGIC receptors. These hydrophobic leucines protrude into the pore and their presence on each of the five subunits enables them to form a pentameric radially symmetrical arrangement of hydrophobic bonds that holds the channel closed. As many functional studies have demonstrated [99-102], substitution of one or more of these leucines with polar or charged residues disrupts some of the bonds, leading to a collapse of symmetry and the conversion of all TM2 domains to the open pore conformation. Thus, one mutated subunit per receptor would be sufficient to cause a significant, potentially damaging, chloride leak current.

Unlike *GLRA1* mutations, *GLRB* mutations are strongly associated with delays in gross motor development and speech acquisition in humans [4]. This fact resembles the situation in zebrafish, where morpholino knockdown of the zebrafish orthologues of *GLRA1* and *GLRB* results in distinct startle phenotypes [103]. The differential effect in mammals may be explained by the fact that β subunits are expressed at a much earlier developmental stage than α1 subunits, where they are involved in the formation of the first glycinergic synapses together with α2 subunits [23,104].

## Conclusions

*GLRA1* and *GLRB* hyperekplexia mutations can be grouped into three main categories. The first includes those dominant mutations located in and around the TM2 domain that do not impair cell surface expression but disrupt hGlyR function by either inducing spontaneous channel activity or by reducing glycine sensitivity, chloride conductance and/or open probability. The second category includes those recessive missense mutations located throughout the receptor that result in a deficiency in cell surface targeting of hGlyRs. The final category includes recessive nonsense and deletion/frameshift mutations, the so called null genotypes, which preclude the formation of full length functional pentamers. The analysis of the molecular mechanisms of these mutations has provided unexpected insights into the structure and function of GlyRs and also into glycinergic signaling mechanisms in health and disease. By describing some of these molecular mechanisms, we hope that we have been able to provide explanations for the phenotypes of many gene-positive patients.

## Abbreviations

hGlyR: Human glycine receptor; nAChR: Nicotinic acetylcholine receptor; pLGIC: Pentameric ligand-gated ion channel; TM: Transmembrane.

## Competing interests

The authors declare that they have no competing interests.

## Authors’ contributions

Both authors participated in developing and discussing the ideas and in writing the manuscript. Both authors have read and approved the final manuscript.
